# Selective cellular and regional vulnerability in frontotemporal lobar degeneration: a scoping review

**DOI:** 10.17879/freeneuropathology-2025-5812

**Published:** 2025-04-09

**Authors:** Kashif Ravasia, Veronica Hirsch-Reinshagen

**Affiliations:** 1 School of Medicine, University of British Columbia, Vancouver, Canada; 2 Division of Neuropathology, Vancouver General Hospital and University of British Columbia, Vancouver, Canada

**Keywords:** FTLD, Selective vulnerability, Human post mortem, Histology, Tau, TDP-43, FUS

## Abstract

The three main types of frontotemporal lobar degeneration (FTLD) are characterized by the accumulation of abnormal proteins, namely tau, TDP-43 and FUS. The distribution of these proteins within different human brain regions is well known, as is the range of morphological variability of the cellular inclusions they form. Compared to the extensive knowledge that exists about distinct protein aggregates in FTLD, surprisingly little is known about the specific cell (sub)types that these inclusions affect. Even less is known about disease-specific abnormalities other than protein inclusions in affected and unaffected areas. These are non-trivial knowledge gaps. First, knowing which cell subtypes are vulnerable or resilient to the development of pathological protein inclusions is crucial to understand the cellular disease mechanisms. Second, mounting evidence suggests that non-cell autonomous mechanisms may play important roles in neurodegenerative conditions. For example, astrocytic tau pathology is associated with synaptic loss in corticobasal degeneration but not in progressive supranuclear palsy. Furthermore, changes that are more difficult and time-consuming to quantify, for example loss of a specific neuronal subtype that does not develop pathological inclusions, remain virtually unexplored and their relevance for disease progression are unknown. This scoping review is an attempt to collate all histological evidence from human studies that address the question of cell-specific vulnerability in the most common FTLD subtypes. By taking a systematic approach including various brain cell types such as neurons and their subtypes as well as astrocytes, microglia and oligodendrocytes and the entire central nervous system with its affected and unaffected regions, this review summarizes the current status in the field and highlights important knowledge gaps.

## Introduction

 Frontotemporal dementia (FTD) is a clinical neurodegenerative syndrome characterized by prominent deficits in behaviour, language, and/or personality, with relative sparing of memory early in the disease course. FTD is the third most common form of neurodegenerative dementia after Alzheimer’s disease (AD) and dementia with Lewy bodies, and affects about 20,000 to 30,000 people in the United States according to estimates by Knopman and Roberts^1^ alone. This prevalence is similar to that identified in the United Kingdom^2^. FTD is phenotypically heterogeneous, including cases of behavioural variant FTD (bvFTD) and primary progressive aphasia (PPA), the latter of which can be additionally subclassified into nonfluent variant PPA (nfvPPA), semantic variant PPA (svPPA), or logopenic variant PPA (lvPPA)^3^. These subtypes can often be difficult to disentangle clinically, as additional symptoms may develop as the disease progresses, and symptoms can mimic or overlap with other neurodegenerative conditions such as AD, Parkinson’s disease (PD), or amyotrophic lateral sclerosis (ALS)^3^. 

 The neuropathological condition underlying most cases of FTD, termed frontotemporal lobar degeneration (FTLD), is also highly heterogeneous. The clinical diagnosis of FTD and the neuropathological diagnosis of FTLD are not always concordant. Patients with pathological FTLD and significant loss of memory may be diagnosed in life with AD, and those with significant motor symptoms may be diagnosed with ALS or PD^4,5^. Conversely, some cases of clinically diagnosed bvFTD and lvPPA meet neuropathological criteria for AD^6,7^. As the name implies, FTLD is characterized by preferential atrophy of the frontal and temporal lobes and abnormal protein inclusions in neurons and glial cells. Historically, the first inclusions to be recognized were argyrophilic Pick bodies in Pick’s disease (PiD)^8^. Until the 1980s, cases with clinical FTD and a FTLD pattern of brain atrophy but without Pick bodies were described as atypical PiD cases^8,9^. With the advent and widespread adoption of immunohistochemistry (IHC), it became possible to identify novel protein inclusions in FTLD cases. The first one to be identified was hyperphosphorylated tau^10,11^, which ultimately led to a revised and expanded categorization of tau-positive FTLD, including PiD. In 2006, another protein, transactive response DNA binding protein 43 (TDP-43), was identified in about 90 % of cases of tau-negative FTLD^12,13^. Most of the remaining cases were later found to contain a protein known as RNA-binding protein fused in sarcoma (FUS)^14,15^. While there remains a small group of cases that stain positive for ubiquitin but negative for tau, TDP-43, and FUS, over 99 % of FTLD cases can now be classified into tauopathies (FTLD-tau), FTLD with TDP-43 pathology (FTLD-TDP), and FTLD with FUS pathology (FTLD-FUS)^15^. Each of these neuropathological entities exhibits known patterns of neuronal and glial inclusions^16^ in the neocortex, deep grey nuclei and infratentorial structures. 

 In the different FTLD pathologies, the selective vulnerability of different brain cell types such as neuronal subtypes, astrocytes, microglia and oligodendrocytes is incompletely understood. Selective neuronal vulnerability has been a focus of recent research on neurodegenerative diseases including AD and PD. In each of these syndromes, specific sets of neurons degenerate more quickly and more consistently than other neuronal populations^19,20^. Furthermore, in each disease affected neuronal populations display similar alterations in organelle distribution, neurotransmitter receptors, electrophysiology, and/or morphology^19,20^. This patterned neurodegeneration has been less studied in FTLD. In addition, FTLD pathology prominently affects glial cells whereas in AD or PD the pathological inclusions are mainly neuronal. The pathophysiological effects of the glial involvement in FTLD are largely unknown. Pathological glial involvement has been shown in amyotrophic lateral sclerosis (ALS), where progressive motor neuron degeneration has been shown to be modulated by non-neuronal cells through a process known as non-cell autonomous neurodegeneration^21,22^. Therefore, studies that seek to understand the processes responsible for patterned neurodegeneration should consider and include the evaluation of non-neuronal dysregulation as one possible pathological mechanism of disease progression. 

Here, we review the available literature on selective neuronal vulnerability in FTLD and include data on glial pathology and its relationship to neuronal pathology wherever possible. The review is organized primarily according to the type of FTLD proteinopathy and the FTD disease subtype. This review places special emphasis on human post mortem studies involving colocalization of pathology using IHC or immunofluorescence, as these studies allow identification of specific cell populations and definitive neuropathological diagnosis of each case. In the IHC studies described below, the most commonly used subtype-specific markers include parvalbumin and calbindin for inhibitory neurons and subtype-specific neurotransmitters or enzymes involved in neurotransmitter metabolism such as choline acetyltransferase (ChAT) or tyrosine hydroxylase (TH). Glial cell-specific markers include glial fibrillary acidic protein (GFAP) and aquaporin 4 (AQP4) as pan-astrocytic markers, and vimentin and CD44 as markers of activated or reactive astrocytes. Microglial markers include cluster of differentiation 68 (CD68), human leukocyte antigen – DR isotype (HLA-DR) and ionized calcium-binding adapter molecule 1 (Iba1).

## 1. Frontotemporal lobar degeneration with tau pathology

 Tauopathies, named for the accumulation of microtubule-associated protein tau, account for approximately 40 percent of FTLD cases^23^. While numerous tauopathies have been described and characterized, this review focuses on the three FTLD tauopathies for which the existing literature is most robust: Pick’s disease (PiD), corticobasal degeneration (CBD), and progressive supranuclear palsy (PSP). 

### 1.1 Pick’s Disease

#### 1.1.1 General features

**Figure 1. Pathological features of Pick’s Disease.**
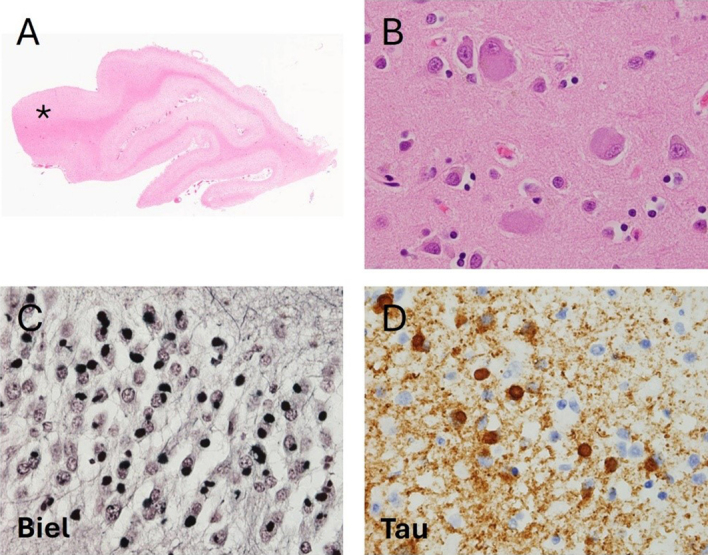
**A**. Low power image reveals relative preservation of the posterior aspect of the superior temporal gyrus (asterisk, 50x). **B**. Hematoxylin and eosin stain reveals neocortical ballooned neurons (600x). **C**. Bielschowsky silver stain reveal numerous argyrophilic round inclusions (Pick bodies) in the granular neuronal cells of the hippocampal dentate fascia (600x). **D**. Tau immunohistochemistry reveals numerous tau-immunoreactive Pick bodies and granular neuropil staining (600x).

 PiD typically presents with bvFTD or lvPPA, and the gross appearance involves frontotemporal atrophy with relative sparing of the Rolandic cortex and the posterior superior temporal gyrus^24^ (**Figure 1**). Neuropathologically, PiD demonstrates astrocytosis, loss of neurons, ballooned neurons with eosinophilic cytoplasm, and extensive spongiosis^25^ (**Figure 1**). Most strikingly andof pathognomic value, PiD demonstrates Pick bodies, i.e. round, solitary, argyrophilic neuronal cytoplasmic inclusions (NCIs) primarily located in pyramidal neurons and in the dentate granular neurons of the hippocampus^26^ (**Figure 1**). Glial pathology has also been observed in PiD cases , including ramified astrocytes and small oligodendroglial inclusions^24^. Pick bodies consistently display 3-repeat (3R) tau pathology, while astrocytes display a variable combination of 4R and 3Rtau^16,25,27,28^, depending on the cases studied. Many of the studies evaluating the selective cellular vulnerability in PiD were conducted before the modern classification scheme for FTLD making it difficult to confirm whether these cases actually correspond to PiD. 

#### 1.1.2 Neuronal pathology

**Table 1** provides a summary of the known neuronal subtypes that bear tau pathology and/or undergo selective neurodegeneration in PiD. Many regions of the frontal and temporal cortex display marked neuronal loss which begins in the outer cortical layers and later progresses to involve the deeper layers of the cortex and extracortical regions^25^. Pyramidal cell density is most markedly reduced in lamina II of the cortex, with lesser involvement of laminae III and V, and occurs in the early stages of the disease^29^. This finding is consistent with studies of laminar distribution of tau pathology in PiD, which have demonstrated that Pick bodies are prominent in laminae II and III, with variable involvement of deeper cortical layers^25,30^. 

**Table 1:** Summary of specific neuronal subtypes susceptible to tau accumulation and/or neurodegeneration by brain region.

**Region **	**Pick’s Disease**	**CBD**	**PSP**
**Neocortex**	Superficial pyramidal cells; von Economo neurons	Deep and small superficial neurons – unknown subtype; loss of excitatory synapses	Corticocortical projection neurons (pre-supplementary motor area); inhibitory interneurons (primary motor cortex); upper motor neurons; loss of excitatory and inhibitory synapses
**Hippocampus**	Dentate granule cells	Dentate granule cells and pyramidal CA2 neurons	parahippocampal gyrus – unknown subtypes; pyramidal CA1 neurons
**Basal ganglia**	Caudate > putamen – unknown subtype	Caudate and putamen > GP – unknown subtype	GABAergic neurons
**Thalamus**	Mediodorsal nucleus – unknown subtype	Ventrolateral portion – unknown subtype	Intralaminar nuclei (centromedian and parafascicular); ventrolateral nucleus
**Brainstem**	Raphe nuclei (5-HT) and LC (NA) > SN; pontine nuclei; DM X	SN – both DA and GABA	SNr (parvalbumin); SNc; ventral tegmental area and the parabrachial pigmented nucleus (TH); LC; mesencephalic motor nuclei; mesopontine nuclei (cholinergic and non-cholinergic cells)
**Cerebellum**	mossy fibers, monodendritic brush cells, dentate projection neurons	Purkinje cells, cerebellar dentate neurons	Purkinje cells, cerebellar dentate neurons

Abbreviations: CBD: Corticobasal degeneration; DA: dopamine; DM X: dorsal motor nucleus of the vagus nerve; LC: locus coeruleus; NA: noradrenaline; SN: substantia nigra; SNr: substantia nigra pars reticulata; SNc: substantia nigra pars compacta; PSP: Progressive supranuclear palsy; TH: tyrosine hydroxylase; 5-HT: 5-hydroxytryptamine or serotonin.

 Von Economo neurons (VENs) are found in lamina V of the anterior cingulate and frontoinsular regions of the cortex and are morphologically distinct from neighboring pyramidal neurons^31^. VENs have been shown to be significantly affected in confirmed PiD cases and degenerated more rapidly than neighboring cells^32^. Moreover, VENs demonstrate tau aggregation and cytoplasmic swelling in all stages of PiD^31^, which is consistent with the observation that early neuron loss and astrocytosis are most significant in the orbitofrontal and mediofrontal cortices in this disease^25,33^. 

 In the hippocampus, the dentate gyrus is most significantly involved in PiD^34^, with greater tau pathology in granule than in hilar cells^35^. One study found a trend toward decreased calbindin immunoreactivity in dentate granule cells in PiD, as compared with other pathological subtypes of FTLD. This trend did not reach statistical significance, and loss of immunoreactivity may also be secondary to generalized neuronal atrophy rather than to selective vulnerability of dentate cells^36^. 

 As early as 1998, it was observed that the striatum can exhibit atrophy in severe or longstanding cases of PiD, with greater involvement of the caudate than the putamen^26^. These findings have been confirmed in a more recent study on the progression of PiD, in which the researchers also detected late developing tau depositions in the globus pallidus^25^. These tau inclusions are primarily intra-neuronal, but the phenotypic features of the affected neurons have not been studied in detail^25^. Limited evidence suggests the mediodorsal nucleus of the thalamus is preferentially affected in PiD^37^. Furthermore, asymmetric thalamic atrophy has been observed by volume-based magnetic resonance imaging (MRI)^38^, but the affected neuronal populations remain unknown. 

 The brainstem can also be neuropathologically affected in PiD. In one study, pontine involvement was found to develop after the onset of limbic and neocortical disease, and the serotonergic and noradrenergic nuclei of the raphe nuclei and locus coeruleus exhibited more severe pathology relative to the remaining brainstem^25^. Involvement of the substantia nigra, the dorsal motor nucleus of the vagus nerve and other brainstem nuclei was observed to coincide with pontine pathology, while involvement of the inferior olivary nuclei and medullary pyramids occurred only in advanced disease stages^25^. There is rare literature on the involvement of the cerebellum in PiD. In 1999, Braak *et al.* determined that mossy fibers, monodendritic brush cells, and dentate projection neurons were involved in a set of cases classified as PiD, but these findings have not been replicated yet^39^. 

In summary, the distribution of tau pathology and neuronal loss in the cortex of patients with PiD seem to be correlated, particularly with regard to VENs. Yet, the relationship between tau pathology and rate of loss of different neuronal populations is still unknown for the remaining brain areas such as hippocampus, brainstem and cerebellum, where the distribution of the PiD tau pathology has been described in detail.

#### 1.1.3 Glial involvement

 PiD cases display significant astrogliosis and astroglial tau pathology in cortical regions. Astroglial tau pathology is most notable in the orbitofrontal cortex and mediofrontal cortices, which show significant neuronal loss and tau-immunopositive ramified astrocytes even in early stages of disease^25^. In early stages of PiD, IHC for GFAP, a marker that is increased in reactive astrocytes, demonstrates widespread astrocytic reaction in laminae I, III, and IV^33^. Some of these astrocytes also stain positive for tau, with one double-labelling IHC experiment showing that 23 % of cortical GFAP-positive astrocytes were also positive for tau and that tau-positive filamentous inclusions can sometimes displace GFAP-positive fibrils^29^. Interestingly, markers of astrocytic apoptosis and dysregulated ceramide metabolism, thought to be neuroinflammatory and pro-apoptotic, have been found in regions of neuronal loss in PiD^33,40^. 

 Oligodendroglial coiled bodies are restricted to areas affected by neuronal tau pathology and degeneration in PiD^41^. Interestingly, a PiD-specific oligodendroglial inclusion has been described^24,41^, but its relationship to oligodendrocyte degeneration or axonal loss is unknown. 

 Similar to astrogliosis, microgliosis has been consistently described in the frontal and temporal cortex of PiD for decades. This finding has been confirmed in cases meeting revised criteria following the development of the modern FTLD classification system^13,18,42^. Cortical microglial cells demonstrate activation by enhanced staining for HLA-DR^29^ and this increased microglial activation is especially notable in areas displaying a high burden of Pick bodies^43^. The extent of grey matter microglial involvement in PiD seems to be quite substantial. Indeed, comparative studies have shown a significative increase in grey matter microgliosis not only relative to controls but also relative to cases of CBD, PSP, and rare tauopathies^42^. These differences are observed regardless of the IHC protocol used to identify microglia, including CD68, Iba1, and CR3/43, a novel antibody that reacts with the human leukocyte antigen isotypes DR, DP, and DQ^42^. 

 Additional studies consistently show microgliosis in the white matter underlying the frontal and temporal cortex^29,42,43^. When activated microglia are measured specifically, whether by using CD68 or HLA-DR, more gliosis is seen in the white matter than in the grey matter^29,42^. By contrast when total microglial burden is measured using Iba1, the difference between these regions is less evident^42^. Microglial dystrophy is also more severe in subcortical white matter than in the cortical grey matter, with many microglia demonstrating a loss of fine branches and unusual cytoplasmic morphology^42^. 

Together, these data suggest that neuronal loss, astroglial reactivity and tau pathology significantly yet not perfectly overlap in the cortex in PiD. How these three alterations relate to each other has not been described in detail. Furthermore, microglial activation and dystrophy seem greater in the white matter than in the overlying cortex. The relationship between microglial alterations, oligodendroglial changes, axonal and neuronal loss as well as tau pathology remains unclear. Finally, despite the early and consistent involvement of the hippocampus, and the known involvement of subcortical structures later in PiD, there are no additional details on glial involvement and their relationship to neuronal tau pathology in these areas.

### 1.2 Corticobasal Degeneration

#### 1.2.1 General features

 Corticobasal degeneration (CBD) is a 4R tauopathy initially described as the underlying pathological condition of a clinical syndrome now termed corticobasal syndrome (CBS). CBS is characterized by asymmetric rigidity and apraxia, dystonia, myoclonus, and cortical symptoms^44^. However, it is now understood that CBD can also present clinically as nfvPPA, progressive supranuclear palsy syndrome (PSPS), frontal behavioural-spatial syndrome (FBS), or the classic CBS motor syndrome^45^. Moreover, cases of clinically diagnosed CBS have been shown on post-mortem pathological examination to meet diagnostic criteria for PSP, PiD, AD, Creutzfeldt-Jacob disease, and FTLD-TDP^48,49^. 

**Figure 2. Pathological features of Corticobasal degeneration and Progressive supranuclear palsy.**
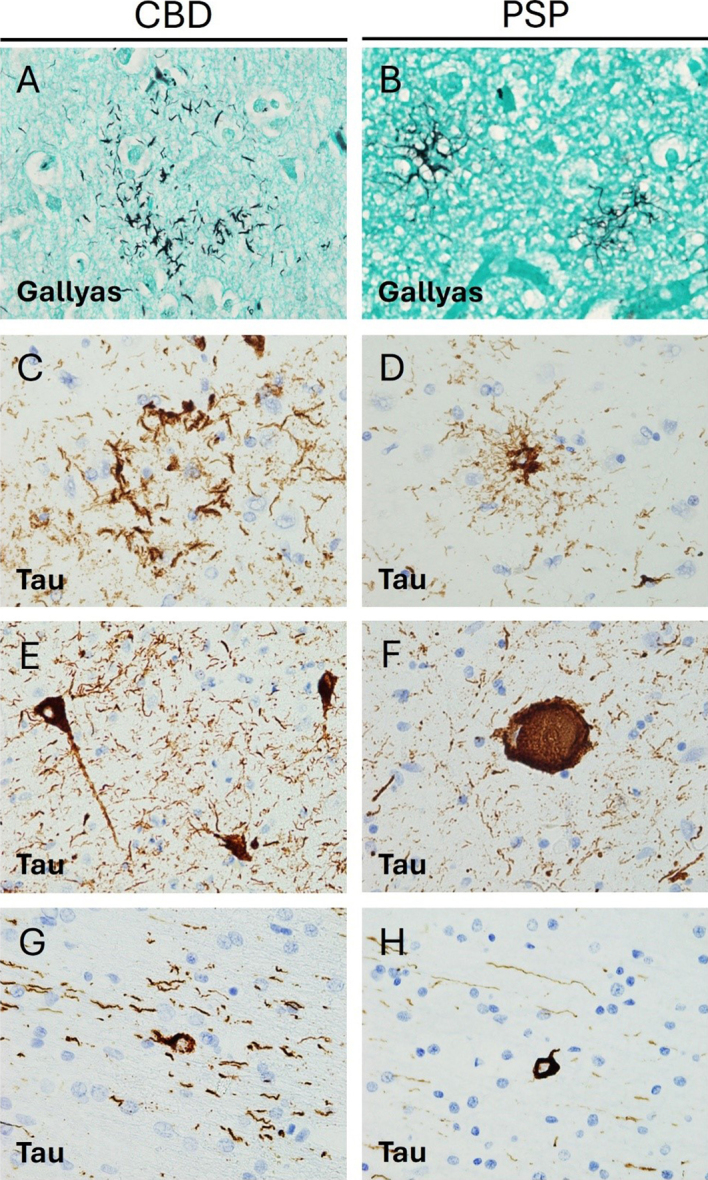
**A, B.** Gallyas staining reveals different argyrophilic astrocytic inclusions. In CBD (**A**) only the distal processes of astrocytes are stained, whereas in PSP (**B**) tufted astrocytes display increased proximal cytoplasmic staining (600x). **C, D.** Tau immunohistochemistry (IHC) reveals differences in astrocytic morphology between CBD and PSP with astrocytic plaques in the former (**C**) and tufted astrocytes in the latter (**D**) (600x). **E, F**. Tau IHC highlights neurofibrillary tangle pathology in both conditions, with globose tangles (**F**) being more frequent in PSP. Note increased background thread pathology in CBD (**C, E**) (600x). **G, H.** Oligodendroglial coiled bodies are seen on tau IHC in both conditions (600x).

 Neuropathologically, CBD is characterized by cortical atrophy that tends to involve the peri- Rolandic cortex but can also involve temporal regions associated with language, and anterior frontal regions associated with behaviour and personality^50^. CBS cases exhibit cortical spongiosis and astrogliosis of the superficial laminae and ballooned neurons in laminae III, V, and VI. Diagnostic tau-immunoreactive pathology includes neurofibrillary tangles, numerous thread-like lesions in white and grey matter^50^ and characteristic glial lesions such as astrocytic plaques and oligodendroglial coiled bodies^51^ (**Figure 2**). 

#### 1.2.2 Neuronal pathology

**Table 1** provides a summary of the known neuronal subtypes that bear tau pathology and/or undergo selective neurodegeneration in CBD. CBD is characterized by atrophy of the frontal, parietal and temporal cortex, with consistent involvement of the premotor cortex^52^. In keeping with the diverse range of clinical presentations, 4R-tau can accumulate in a variety of neuroanatomical regions. In classical CBS cases, 4R-tau shows peri-Rolandic distribution^46^, but there are also cases with predominant temporal involvement^46,53^ and some with an unusual degree of frontal tau pathology^53^. Cortical neurons demonstrate ballooning and achromasia in deep cortical layers and a variety of tau immunoreactive pathology ranging from granular pre-tangles to more filamentous neurofibrillary tangles^51,54^. Small neurons in upper cortical layers are most vulnerable to CBD^51^. In some cases of CBD, hippocampal neurons in the CA2 region and the dentate gyrus can show tau pathology ^50^. The extent of tau pathology varies by clinical presentation, being more pronounced in PSPS than in CBS despite pathological confirmation of CBD in both sets of cases^46^. 

 CBD also exhibits extensive pathology in the basal ganglia. Filamentous neuronal inclusions are often visible in the caudate and the putamen, but less consistently seen in the globus pallidus^51^. Neuronal pathology tends to become more severe with disease progression^49,52^. Confirmed cases of CBD often exhibit mild-to-moderate neuronal loss and tau-positive neuronal inclusions and threads in the thalamus^55^, which is consistently affected in its ventrolateral portion^56^. 

 In CBD, the brainstem is not classically considered as a region of interest, but the substantia nigra can be affected. In these cases, the substantia nigra appears markedly depigmented with pale intracytoplasmic inclusions in surviving neurons^57^, numerous pre-tangles, and severe loss of dopaminergic and GABAergic neurons but without significant astrocytic plaque pathology^51,58,59^. Some CBD cases also show tau deposition in the tegmentum and inferior olivary nucleus. This phenomenon has not been studied in detail, but current evidence suggests that medullary tau deposition is more common in cases that clinically present as PSPS^46^. 

 The cerebellum has not been extensively studied in CBD, although variable neuronal loss and gliosis were found in the cerebellar dentate nucleus together with scattered cortical Purkinje cell axonal torpedoes and mild Bergmann gliosis^51^. One study found the cerebellum to be involved in approximately half of the cases studied^60^. Cerebellar involvement mainly consisted in diffuse granular accumulation of cytoplasmic tau in the cell bodies of Purkinje cells, and of doughnut-shaped structures in the cerebellar molecular layer in a smaller set of cases^60^. While the latter alterations were not studied directly in any CBD case, ancillary studies in PSP cases revealed their location in the GFAP-positive radial processes of Bergman’s glia^60^. 

A relatively detailed map of neuronal tau-positive pathology has been described in CBD, but the degree of correlation between tau-positive pathology and stereotactically measured neuronal loss remains uncertain, especially in subcortical and infratentorial regions. It also remains unclear whether tau-positive pathology predominates in specific subtypes of affected neurons. It would also be of particular interest to understand these relationships in early and later disease stages.

#### 1.2.3 Glial involvement

 Glial pathology in CBD is of diagnostic significance^51^. Indeed, the characteristic thread pathology of CBD is likely predominantly glial rather than neuronal, as only a small fraction of thread-like structures are double labeled with neurofilament antibodies^41^. Moreover, studies of astrocytic tau pathology provide evidence of glial cell involvement in regions that are preferentially affected by CBD. Astrocytic plaques have been shown to co-localize with CD44, possibly suggesting a reactive change^61^, but not with GFAP^62^. Furthermore, the presence of astrocytic plaques in specific areas correlates with neuronal loss and reduced local density of HOMER1+ excitatory post-synaptic puncta, providing evidence for a relationship between glial and neuronal pathology^63^. One study has described tau-positive astrocytic plaques in the superior frontal gyrus prior to the development of symptomatic neurodegeneration^64^, and another study demonstrated that astrocytes and neurons in the grey matter of the anterior frontal lobe demonstrate tau pathology even in preclinical CBD^65^. A semiquantitative score for astrocytic plaque density was shown to remain moderate throughout disease progression, while the density of neuronal inclusions in the anterior frontal grey matter increased with disease progression^65^. Taken together, these findings have led some scholars to speculate that CBD is a primary astrogliopathy^65,66^. 

 Astrogliosis and astrocytic plaques have also been observed in the hippocampus^67^, and are consistently found in the basal ganglia of confirmed CBD cases. Astrocytic plaques can be found throughout the striatum, although they are more numerous in the caudate than the putamen^65^. Indeed, the caudate is even more severely affected than the anterior frontal gyrus in preclinical CBD^65^. It is also worth noting that these astrocytic plaques develop early in the disease process, and one study has found that basal ganglia astrocytic pathology is most severe in the preclinical stage of CBD, with diminished density of plaques in end-stage disease^65^. These findings suggest an early involvement of basal ganglia astrocytes, in keeping with the frequently observed clinical picture^49^. Astrocytic plaques have also been described in the thalamus, although they are less frequent than in the neocortex or the caudate^58^. Finally, GFAP-positive radial processes of Bergman’s glia^60^ may show doughnut-shaped tau-positive structures in the cerebellar molecular layer in a small set of CBD cases^60^. 

 Oligodendroglial coiled bodies are distributed extensively throughout affected areas in CBD, but they are less frequent than in PSP^41^. Furthermore, little is known about the relationship between these oligodendroglial coiled bodies and other histological aspects such as myelin density or axonal density in the surrounding area^68^. 

 Microglial activation is observed in confirmed CBD cases when assessed by CD68 immunoreactivity^42^. In one large comparative study of frontotemporal microglial burden, CD68-positive microglia were significantly more numerous in the frontal grey matter than in the temporal grey matter of CBD cases. There was however no significant increase in the density of CR3/43- or Iba-1-immunoreactive microglia^42^. Additionally, the parietal somatosensory and the superior temporal cortex demonstrate more widespread microgliosis in CBD than in PSP^56^. White matter microgliosis is a consistent finding in CBD^56^. Significant differences between controls and CBD cases have been observed in subcortical white matter in the frontal, temporal, and parietal lobes^42,56^. The frontal and temporal subcortical regions display moderate-to-severe microglial dystrophy that is more noticeable in the white matter than in the associated cortical grey matter^42^. Activated microglia are also widely distributed throughout the basal ganglia, with HLA-DR immunostaining demonstrating their significant proliferation in the striatum, the lentiform nucleus, the subthalamic nucleus, and the substantia nigra, as compared to controls^56^. Microglial activation is also observed in the ventrolateral portion of the thalamus^56^. 

The early and region-specific presence of astrocytic pathology as well as the link between astrocytic and synaptic pathology raises the possibility that astrocytic tau pathology may be pathogenic in CBD. It is therefore somewhat surprising that no more detailed studies exist correlating subtype-specific neuronal loss with astroglial tau pathology. Notably, neuronal tau pathology has been described in areas without significant astrocytic pathology such as the brainstem, suggesting that different pathomechanisms may be at play in these regions. Microglial reactivity seems to mirror neuronal pathology in CBD. It endeavors to further dissect the relationship between astrocytic and microglial pathology to understand whether microglial activation reflects a primary neuroinflammatory mechanism or a specific response to neuronal injury.

### 1.3 Progressive Supranuclear Palsy

#### 1.3.1 General features

 PSP is also a 4R tauopathy and presents most often as a movement disorder, yet cognitive decline is quite common and can be the presenting feature^45^. While the classic PSPS involves vertical gaze palsy, unprovoked falls, akinesia, and cognitive dysfunction, each of these symptoms can present along a spectrum of severity, and some cases with predominant akinesia or cognitive involvement are clinically diagnosed as CBS or PPA^69,70^. 

 Neuropathologically, PSP is characterized by globose neurofibrillary tangles, thin, branching astrocytic tau inclusions (“tufted astrocytes”), and oligodendroglial coiled bodies (**Figure 2**). The basal ganglia and brainstem tend to be especially involved, though cases with features suggestive of frontotemporal dementia often demonstrate substantial cortical involvement^8^. 

#### 1.3.2 Neuronal pathology

**Table 1** provides a summary of the known neuronal subtypes that bear tau pathology and/or undergo selective neurodegeneration in PSP. Historically, PSP was thought to be primarily a disease of the basal ganglia and the midbrain with cortical involvement being limited and largely confined to the pre-central gyrus^71^. More recent studies have however challenged this view. Indeed, many PSP cases demonstrate widespread frontal and temporal atrophy, and these cases often manifest clinically with cognitive, behavioural, and linguistic symptoms similar to those observed in other forms of FTLD^72,73^. Immunohistochemical studies have demonstrated tau-positive neuronal tangles and neuropil threads in the superior frontal gyrus, middle frontal gyrus, and inferior temporal gyrus, with greater cortical tau pathology in cases that clinically manifest with frontotemporal dementia as compared to classical PSPS^74^. Analyses using confocal microscopy have shown that both excitatory and inhibitory cortical synapses are reduced in pathologically confirmed cases of PSP with frontal tau pathology^63^. In contrast to CBD, astrocytic pathology in PSP does not appear to correlate locally with loss of synapses^63^, suggesting a possible divergence in the mechanisms of synaptic vulnerability between the two diseases. 

 While many of the classical motor deficits in PSP are related to subcortical pathology, the primary motor cortex and supplementary motor areas are often affected in PSP as well. Corticocortical projection neurons in the pre-supplementary motor area and inhibitory interneurons in the primary motor cortex have been identified as particularly vulnerable populations in PSP^75^, but pyramidal neurons also display variable degrees of pathology^76^. Compared to cases presenting clinically with CBS or PPA, cases presenting with classical PSPS have been found to exhibit greater pyramidal motor neuron involvement^76^. 

 Hippocampal involvement in PSP remains poorly characterized in the literature. Preliminary studies have demonstrated enlarged neurons and neurofibrillary tangles in the parahippocampal gyrus (PHG) and in the CA1 sector of the hippocampus^77^. Neuronal tau pathology appears to precede astroglial or oligodendroglial involvement in the hippocampus, and the burden of neuronal pathology can be quite severe^78^. 

 By contrast, basal ganglia have been studied extensively in PSP with consistent findings of early neuronal and glial tau pathology throughout the striatum, globus pallidus, and subthalamic nucleus^8^. GABA is the primary neurotransmitter involved in basal ganglia circuitry and decreased expression of GAD-67, a marker of GABAergic interneurons, has been confirmed in case-control studies^79^. However, the relationship between neurofibrillary tau pathology and affected neuronal subtypes has not been evaluated. Additionally, the nucleus basalis of Meynert exhibits mild-to-moderate neuronal loss and a reduction in ChAT positivity has been shown in at least some cases. Altogether however, the basal forebrain is only modestly affected in PSP in comparison to other neurodegenerative conditions^80^. 

 PSP cases demonstrate both neuronal loss and microglial activation in many regions of the thalamus. In particular, the intralaminar nuclei appear to be profoundly affected, with one case-control study reporting a loss of 45 % of neuronal density across the centromedian and parafascicular nuclei in PSP cases^81^. The ventral lateral nucleus also exhibits atrophy and neuron loss, particularly in cases with greater involvement of the primary motor cortex^75^. 

 The brainstem exhibits striking changes in PSP. Typically, both divisions of the substantia nigra are affected. In the pars reticularis (SNr), there is a loss of overall neuron density and decreased parvalbumin reactivity among surviving neurons, suggesting particularly pronounced vulnerability among the parvalbumin-positive cells^82^. This selective involvement of circuits involving parvalbumin-positive neurons has also been observed in Parkinson’s disease (PD). Yet, PSP cases appear to exhibit more severe disruptions to the parvalbumin-positive interneurons and more frank atrophy than PD cases^82^. 

 In the pars compacta (SNc), there is a duration-dependant, selective dropout of neuromelanin-positive cells^82^. Indeed, for reasons that are poorly understood, dopaminergic cells appear profoundly vulnerable to the changes induced by PSP. Tyrosine hydroxylase-immunoreactive cells (TH-IR) in the nearby A10 region including the midline ventral tegmental area and the parabrachial pigmented nucleus are also affected with the loss of approximately 50 % of TH-IR neurons compared with controls^83^. Disruptions to dopaminergic signalling have been experimentally linked to downregulation of parvalbumin circuitry in mouse models, providing a potential explanation for the selective vulnerability of these two distinct neuronal populations^84^. 

 PSP cases also often demonstrate marked but selective neuronal loss in the locus coeruleus and the mesencephalic motor nuclei^85,86^. The locus coeruleus displays marked loss of noradrenergic neuromelanin-positive neurons explaining the relative pallor visible on gross inspection. Quantification using IHC has revealed a loss of 49 % of neuromelanin-positive neurons relative to controls^86^. Cholinergic neurons in the mesopontine nuclei, including the lateral dorsal tegmental nucleus and the pedunculopontine nucleus (PPN) are also affected^80^. More recently, these results have been replicated in a case-control study conducted by Sébille et. al. (2019), showing that PSP cases exhibit greater neuronal loss than controls in both the PPN and the cuneiform nucleus^85^. Both cholinergic neurons, identified using IHC for ChAT, and non-cholinergic neurons are affected, and the PPN is more severely affected in PSP than in PD^85^. Notably, the study found minimal neuronal loss in the surrounding regions^85^, supporting the hypothesis that disease propagation is not driven by anatomical proximity alone. 

 There is evidence to suggest moderate involvement of the cerebellum in most clinical phenotypes of PSP^78^. There is also increasing awareness of a rare clinical presentation of PSP with predominant cerebellar ataxia, which tends to exhibit more pronounced cerebellar neuron loss, tau-positive granular profiles in Purkinje cells, and grumose degeneration in the dentate nucleus^87^. 

Neuronal involvement has been evaluated in greater detail in PSP than in CBD and PiD. It is interesting to note that inhibitory neurons seem to be affected at least in the cortex, basal ganglia, and substantia nigra in PSP. In addition, other neuronal subtypes such as cholinergic and dopaminergic also seem affected, suggesting that neurotransmitter subtype is not the defining feature of vulnerable neurons to PSP pathology. Based on this, transcriptomic studies may provide additional insights into the similarities of vulnerable neuronal subpopulations in PSP.

#### 1.3.3 Glial involvement

 Astrocytic pathology in PSP is complex and intriguing, as there is not always a direct relationship between astrogliosis and the presence of tufted astrocytes. For example, one study showed that despite a very substantial burden of tau-immunoreactive tufted astrocytes in the motor cortex, many cases exhibit only minimal gliosis when evaluated with GFAP^88^. This finding cannot be attributed to variation between cases, as the cases demonstrated remarkable homogeneity in the pattern of gliosis. Tufted astrocytes, similar to astrocytic plaques, have been shown to co-localize with CD44^62^, possibly suggesting a reactive change^61^, but not with GFAP. 

 Some research has been conducted into the involvement of astrocytes and oligodendrocytes in the subcortical white matter, and no significant difference has been demonstrated in the burden of GFAP or myelin basic protein (MBP) between PSP cases and controls^89^. Curiously, one biochemical study found that insoluble tau was detectable in white matter regions by Western blotting despite the absence of tau immunostaining in contiguous sections^90^. Finally, evaluation of oligodendrocyte-specific pathology suggests that PSP is not a primary oligodendrogliopathy, in contrast to multiple system atrophy and globular glial tauopathy^91^. 

 Astrocytic pathology in the basal ganglia appears to be an early event, with particularly severe early astrocytic involvement in the striatum^78,92^. The discrepancy between GFAP distribution and astroglial tau pathology has also been described in the basal ganglia. In one study, the caudate and putamen exhibited the highest burden of tufted astrocytes, while the globus pallidus and substantia nigra exhibited most astrogliosis^88^. No relationship has been found between astrogliosis or neuronal tau pathology and tufted astrocyte density. Yet, the severity of astrogliosis has been shown to correlate with the density of neurofibrillary tangles^88,93^. 

 Characteristic astroglial and oligodendroglial inclusions, i.e. tufted astrocytes and coiled bodies, respectively, are consistently found in the thalamus of moderate-to-severe PSP cases. Conditional probability analyses suggest that in most cases thalamic glial inclusions occur later than striatal inclusions but earlier than neocortical inclusions^78^. Analysis of astrocytic pathology has revealed both astrogliosis and tufted astrocytes in midbrain regions, including the tectum and the red nucleus^88^. Astroglial pathology appears more limited in the pons and medulla, with mild astrogliosis and very few tufted astrocytes^78,88^. 

 Microglial activation has also been demonstrated in PSP cases relative to controls. In PSP, the frontal cortex exhibits statistically significant microgliosis that can be detected using immunostaining for HLA-DR or Iba-1^42,56^. The microgliosis is often most severe in the motor cortex^56^, and microglial pathology in this region correlates with neuronal pathology in the same region^56^. Interestingly, the somatosensory cortex also exhibits statistically significant microgliosis^56^. While involvement of the neocortex in PSP is not as pronounced as in CBD^42,56^, the presence of microglial activation suggests that it may be worthwhile to more thoroughly investigate cortical pathology in PSP. 

 PSP cases often demonstrate white matter microgliosis, but the extent and distribution of the latter vary widely across studies. Evidence of increased overall microglial density is conflicting, with one study finding an increase in Iba-1 positive microglia in the frontal white matter and another study finding no increase in Iba-1 positive cells despite an increase in CD68 positivity^42,89^. Evidence of activation is also conflicting, as some studies but not other ones have demonstrated significantly increased microglial burden in the frontal and temporal white matter when assessed with immunostaining for CD68 and HLA-DR and compared with controls^42,43,56^. 

 In the basal ganglia, microgliosis can be extensive, with significant elevations in HLA-DR-positive microglial burden throughout the globus pallidus and subthalamic nucleus^56^. Microglial activation in the thalamus also appears to be widespread when assessed using HLA-DR immunostaining, with increased burden in the ventrolateral nucleus and anterior nucleus when compared to controls or CBD cases. Adjacent structures, such as the mammillothalamic tract and the thalamic fasciculus, also demonstrate increased microglial burden^56^. Finally, the brainstem shows robust microglial activation in the superior colliculus, the medial longitudinal fasciculus, the substantia nigra, the red nucleus, and the pontine base as measured by HLA-DR expression^56^. 

Overall, there is robust knowledge on the distribution of astroglial and microglial activation in PSP. Less understood is the relationship between astrocytic tau pathology and astrocyte reactivity and how these latter relate to microgliosis. There seems to be at least some correlation between neuronal tau pathology and microgliosis. The relative independence of neuronal tau pathology and microgliosis from astrocytic pathology, as exemplified in the brainstem, is intriguing and requires further exploration. In addition, detailed glial transcriptomic phenotyping may help to identify the astrocytic populations responsible for regional astrogliosis and those most vulnerable to accumulation of 4R-tau. Yet, it is also possible that astrocytes undergo proteomic changes and loss of GFAP positivity as tau accumulates. This highlights the need for additional studies evaluating glial involvement in PSP and its relationship to neurodegeneration.

## 2. Frontotemporal lobar degeneration with TDP-43 pathology

**Figure 3. TDP-43 immunohistochemical features of FTLD-TDP subtypes.**
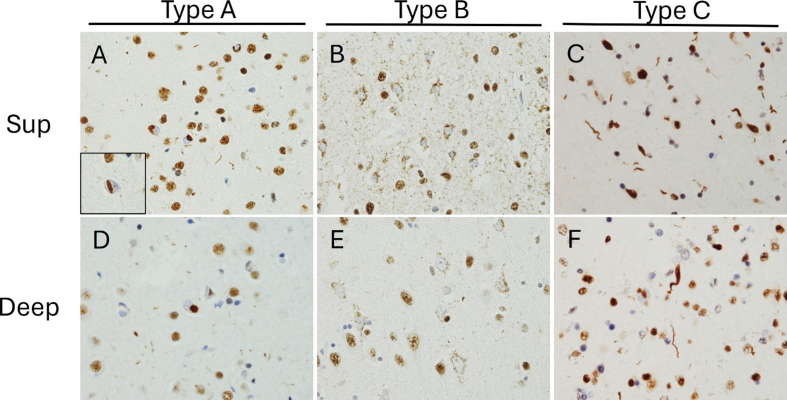
**A,D**. FTLD-TDP type A shows compact neuronal cytoplasmic inclusions (NCIs) and short neurites that predominate in superficial over deep neocortical layers. Neuronal intranuclear inclusions (inset in A) are a distinguishing feature (600x). **B, E.** Type B pathology shows frequent granular NCIs that affect all neocortical layers (600x). **C, F**. Type C pathology is identified by long, frequently corkscrew-like, neuritic inclusions that preferentially affect superficial cortical layers, but can also affect the deep cortex (600x). Sup = superficial.

 TDP-43 was identified in 2006 as the pathological protein present in most cases of ubiquitin-positive, tau-negative FTLD^12,13^. As a result of this discovery, cases that had previously been described as FTLD-U (for ubiquitin) were reclassified as FTLD with TDP-43-immunoreactive pathology (FTLD-TDP). A harmonized histologic classification system for FTLD-TDP now exists with four well-defined subtypes lettered A-D and a more recently discovered, rapidly progressive phenotype provisionally labelled “type E”^94,95^. FTLD-TDP type D is very rare and only found in familial cases with a mutation in the valosin-containing protein (VCP) gene^94^. FTLD-TDP type E is also rare and considered by some authors as a variant of type B^96^. The present review will focus on types A, B, and C, as they collectively account for the significant majority of FTLD-TDP cases^8,94^ (**Figure 3**). 

### 2.1 FTLD-TDP type A

#### 2.1.1 General features

 Clinically, FTLD-TDP type A often presents as bvFTD or nvPPA, though there can also be motor neuron involvement^8^. An heritable form of FTLD-TDP type A caused by a mutation in the progranulin gene (*GRN*) on chromosome 17, and the familial variant often demonstrates more widespread pathology and a younger age at death^97,98^. Moreover, another subset of FTLD-TDP cases is associated with a pathological *C9orf72* hexanucleotide repeat expansion on chromosome 9. Most of these cases fulfill histological criteria of type B, or an overlap between type A and type B (type AB). Only a minority present histologically as pure type A^99^. 

 FTLD-TDP type A is characterized by moderate or numerous compact neuronal cytoplasmic inclusions (NCIs) and dystrophic neurites in layer II of the neocortex^94^ (Figure 3). Lentiform neuronal intranuclear inclusions (NIIs) are also commonly identified, but they are usually much less numerous than NCIs^100^. Late-stage disease often results in neuronal death, loss of positivity for neuronal IHC markers such as NeuN, and, importantly, clearance of inclusions^101^. This can complicate research findings, as TDP-43 positivity can be lost in end-stage disease, necessitating qualitative assessment or adjunctive immunostaining in addition to quantification of pathological inclusions.

#### 2.1.2 Neuronal pathology

 FTLD-TDP type A demonstrates extensive cortical atrophy and proteinopathy with prominent involvement of the superficial cortical laminae. There is also evidence of deeper involvement, with many cases exhibiting short dystrophic neurites and compact NCIs in the deeper cortical laminae^99^. Cases with low levels of overall TDP pathology demonstrate inclusions in projection neurons and in oligodendrocytes of the orbital gyrus and gyrus rectus^102^. When the overall burden of TDP pathology increases during the disease progression, inclusions are found in the middle frontal gyrus, anterior cingulate gyrus, and insular cortex, as well as the superior and middle temporal gyri^102^. These findings provide support for an early involvement of the frontal and temporal lobes. This study however only included bvFTD cases and did not distinguish between type A and type B. To identify the type of neuron affected by neurodegeneration in FTLD-TDP, a recent study compared the postmortem tissue RNA-seq transcriptomes from the frontal cortex, temporal cortex, and cerebellum of 28 control and 30 FTLD-TDP cases. The analysis showed that neuronal loss in the cortex mainly concerns excitatory neurons^103^. Unfortunately, this study did not include pathological subclassification of FTLD-TDP cases in its analysis. 

 As in PiD, VENs appear to exhibit selective vulnerability in FTLD-TDP^104^. Additionally, a population of pyramidal cells neighboring VENs, which likewise express the GABA receptor subunit theta (GABRQ) seem vulnerable in FTLD-TDP. The loss of GABRQ-expressing neurons appears to be correlated with the development of behavioural symptoms at least in FTLD-TDP and FTLD-FUS^104^. Among cases with a *C9orf72* repeat expansion, type A cases appear to demonstrate particularly severe loss of VENs and neighboring GABRQ-expressing pyramidal cells^104^. FTLD-TDP cases with *GRN* mutations have also been shown to exhibit decreased densities of GABRQ-expressing cells^104^. Whether VENs are involved in sporadic cases of FTLD-TDP type A remains poorly understood. While some studies have examined sporadic bvFTD^105,106^, few of the cases examined were clearly type A. 

 FTLD-TDP type A often affects the hippocampus, where the burden of disease can be quite severe. The pathological findings frequently meet criteria for hippocampal sclerosis, defined as “severe hippocampal neuronal loss and gliosis”^99,107^. In the hippocampal dentate gyrus, FTLD-TDP type A cases exhibit a moderate burden of compact NCIs, though they demonstrate a significantly lower burden of compact NCIs than type B or C cases^99^. Unlike other types of FTLD-TDP, type A cases also often exhibit NIIs in the dentate gyrus. Additionally, these cases consistently have delicate wispy threads in CA1, a change that is frequently associated with hippocampal sclerosis. This feature was found to be 100 % sensitive and specific for type A cases in one study^99^. Additional studies are needed to address whether these pathological features show selective predilection for specific hippocampal neuronal subpopulations. 

 The basal ganglia are often involved in cases of FTLD-TDP type A. Long *et al.* have recently suggested that the degree of atrophy in the bilateral caudate and right putamen can distinguish between types A and B with high sensitivity and specificity^108^. These findings are consistent with previous research on the extracortical distribution of dystrophic neurites and neuronal inclusions in FTLD-TDP subtypes. Here, a significant difference was found between dystrophic neurite density in the putamen of type A cases when compared with type B^109^. Furthermore, both FTLD-*GRN* and FTLD-TDP type A without mutations in progranulin have been diagnosed on autopsy in cases of clinical CBS^110^, suggesting that involvement of the striatum can be widespread and clinically significant. 

 Analysis of striatal neuron populations using anti-calcineurin antibodies has revealed marked loss of substance-P positive efferents to the substantia nigra and the globus pallidus pars interna^111^. Enkephalin-positive efferents to the globus pallidus pars externa were also affected, though not as severely, and ChAT-positive striatal interneurons were mostly unaffected^111^. The affected areas also exhibited proliferation of GFAP-positive astrocytes, and the severity of neuron loss was correlated with both the accumulation of phosphorylated TDP-43 inclusions and clinical cognitive symptoms^111^. While these findings have yet to be replicated in a large study powered to distinguish between subtypes of FTLD-TDP, they suggest a selective vulnerability of substance-P-positive striatal efferents. 

 Thalamic atrophy is a relatively common finding in FTLD. MRI findings have suggested that FTLD-TDP cases exhibit greater thalamic involvement than FTLD-tau or FTLD-FUS^112^. In particular, FTLD-TDP type A cases demonstrate significant atrophy in most nuclei of the thalamus when compared to controls, with the possible exception of the ventral posterolateral, ventral medial, pulvinar, and medial geniculate nuclei, where only a trend has been demonstrated^37^. However, more research is necessary to replicate these findings at the histological level and to explore selectively vulnerable populations since only a minority of studies on FTLD-TDP include thalamic histopathological data^113^. 

 Research into brainstem involvement in FTLD-TDP has been limited, and most studies do not explicitly distinguish between subtypes of FTLD-TDP. One study found evidence for dystrophic neurites and multiple types of NCI in the substantia nigra, without involvement of the hypoglossal nucleus^99^. Another small study found that the superior colliculus was involved in all subtypes of FTLD-TDP, and that the substantia nigra, red nucleus, and raphe nuclei also consistently exhibited TDP-43-positive inclusions^114^. More studies are needed to dissect the corresponding cellular details. 

 The extent of cerebellar involvement in FTLD-TDP type A is currently uncertain. Although imaging studies of patients with FTLD-*GRN *fail to demonstrate significant cerebellar atrophy^115^, it is unclear whether this is also the case for sporadic FTLD-TDP type A cases. This may be partly explained by the lack of cerebellar TDP-43 accumulation apparent on histology even in very late stages of disease progression^116^. 

Together, the current information on vulnerable neuronal populations in FTLD-TDP type A points towards selective involvement of excitatory and GABRQ-expressing cortical neurons, substance P-secreting neurons, and to a lesser extent also enkephalin-positive striatal neurons. In the rest of the brain, the distribution of neuronal TDP-43 pathology has been well described, but the degree of subtype-specific neuronal loss relative to this pathology remains unexplored.

#### 2.1.3 Glial involvement

 Astrocytic pathology has not been well-studied in FTLD-TDP type A. Some FTLD-TDP type A cases exhibit glial cytoplasmic inclusions, though they do not appear to be as abundant as in FTLD-TDP type B^99,117^. These glial cytoplasmic inclusions appear to localize primarily to oligodendrocytes in the white matter based on morphology and IHC evidence^117^. Other than the above, immunohistochemical evaluation of astroglial and oligodendroglial involvement in the brain has been largely neglected with the exception of the identification of increased GFAP reactivity in the basal ganglia in general FTLD-TDP^111^ and thalamus in FTLD-GRN^118^. Of promise, conventional IHC and mass spectrometry analysis of the insoluble proteome in FTLD-TDP cases has recently identified a unique pattern of astrocytic F-box protein 2 (FBXO2) expression specifically in type A cases. Further studies will however be required to clarify the proteomic alterations and distribution of these FBXO2-positive astrocytes^119^. Overall, the extent of macroglial involvement in FTLD-TDP type A and its significance for pathogenesis remains largely unexplored. 

 Microglial activation, assessed using CD68 immunostaining, has been shown to be increased in the superficial cortical laminae I-III in FTLD-TDP type A^43^. While the genetic and histological FTLD-TDP classifications do not perfectly match, cases of FTLD with a *GRN* mutation have been shown to exhibit more superficial cortical microglial activation than cases with a *C9orf72 *expansion. This suggests that inherited forms of FLTD-TDP type A due to *GRN* mutations may involve greater microglial activation in affected grey matter regions than inherited type B or AB forms due to *C9orf72 *expansion^120^. The RNA-seq analysis described above also showed that increases in microglial and endothelial cell expression were highly correlated with neuronal loss^103^. The frontal and temporal white matter both display consistent microglial activation when assessed morphologically or by positivity for CD68^42,43^. Historically, it was hypothesized that cases with a *GRN *mutation may exhibit more microglial activation based on the inflammatory functions of progranulin. Yet, post-mortem studies have found no difference between FTLD-TDP cases with or without *GRN* mutations^43^. One study which included mostly type A cases but did not separately analyze results by subtype, found that microglial activation in the hippocampal white matter is significantly greater in FTLD-TDP cases than in controls^121^. In the CA1 region, however, no significant difference was found between FTLD-TDP cases and controls. In the dentate gyrus, the same study found that FTLD-TDP cases actually exhibited less microglial activation than controls^121^. In the thalamus of FTLD-*GRN *cases, Iba-1-positive microglia and GFAP-positive astrocytes were increased in number while myelination measured by myelin basic protein IHC was decreased^118^. 

To conclude, in FTLD-TDP type A microglial activation appears to largely mirror neuronal pathology and loss. There is however not enough available data to draw significant conclusions on the distribution and severity of macroglial involvement in FTLD-TDP type A and its potential pathophysiological relevance.

### 2.2 FTLD-TDP type B

#### 2.2.1 General features

 FTLD-TDP type B is characterized by at least moderate numbers of NCIs throughout all layers of the cortex^94^. These inclusions usually have a diffuse granular morphology, in contrast to the compact elliptical or crescentic inclusions found in type A^100^ (**Figure 3**). Clinically, FTLD-TDP type B usually presents as bvFTD or as motor neuron disease plus FTD, with a significant clinical and genetic overlap between FTD and ALS^94^. 

 There are both sporadic and genetic forms of FTLD-TDP type B, and the genetic causes usually overlap with genes implicated in the pathogenesis of ALS. FTLD-TDP types B or AB are most frequently associated with pathological *C9orf72* hexanucleotide repeat expansions, and particularly prone to manifest with psychotic symptoms and motor neuron disease (MND)^100^. However, it is important to recognize that the association between type B pathology and psychotic symptoms is not entirely explained by the effect of *C9orf72* expansions, as sporadic cases with type B pathology also display an increased frequency of psychotic symptoms^96^. Patients with *C9orf72* mutations produce aggregation-prone dipeptide repeat (DPR) proteins including glycine-alanine (poly-GA), glycine-arginine (poly-GR), proline-alanine (poly-PA), proline-arginine (poly-PR), and glycine-proline (poly-GP), due to unconventional translation of the abnormal hexanucleotide repeat expansion in *C9orf72*^122^. In these patients, the DPRs are found throughout the brain, with the cerebellum exhibiting the greatest concentration of total and soluble DPRs^123^. This review will not explore region-specific expression of dipeptide repeat proteins as a definite positive association between DPR burden and disease-associated clinical symptoms has not been established.

#### 2.2.2 Neuronal pathology

 The predominance of early behavioral symptoms in FTLD-TDP type B suggests that the orbitofrontal cortex and/or limbic structures are affected early during disease. Evidence to support this hypothesis comes from data-driven machine staging models^116^ and retrospective chart reviews after definitive diagnosis^124^. However, familial forms of FTLD-TDP can have different patterns of clinical and pathological progression, and these differences can be even more pronounced in comparison to sporadic cases^23^. 

 As described for type A, a transcriptomic study^10,2^ showed evidence of selective loss of excitatory cortical neurons in FTLD-TDP, but this study did not include FTLD-TDP subtyping^103^. An earlier study from 1993 on cases with FTLD-TDP associated with ALS showed significant decreases in calbindin positivity without changes in expression of parvalbumin, suggesting that neurons expressing calbindin may be affected to a greater degree than neighboring neurons^125^. Yet, this study was performed before the discovery of TDP-43 and thus predated the modern classification of FTLD-TDP. 

 Like FTLD-TDP type A cases, type B cases often involve VENs of the anterior cingulate cortex. As described by Nana *et al.*, even early-stage cases appear to exhibit disproportionate cytoplasmic TDP-43 immunopositivity in VENs, and the accumulation of TDP-43 pathology in this neuronal population correlates with clinical severity^126^. Additionally, fork cells, which are found in the frontal insula alongside VENs, have been found to display a similar pattern of early neurodegeneration^126^. In both VENs and fork cells, cells displaying inclusions or nuclear TDP-43 depletion develop somatodendritic atrophy, suggesting direct neurotoxicity^126^. While some of the FTLD-TDP type B cases studied had *C9orf72* repeat expansions, most were sporadic. Further evidence for the involvement of VENs in sporadic FTLD-TDP type B comes from earlier studies on sporadic bvFTD, in which many cases were either unclassified or type B^105,106^. 

 In the hippocampus, FTLD-TDP type B cases demonstrate a greater burden of both compact and diffuse NCIs in the dentate gyrus and in the CA1 region of the hippocampus compared to FTLD-TDP type A^99^. However, FTLD-TDP type B cases do not demonstrate hippocampal NIIs, dystrophic neurites, or threads, and as a result, the total TDP-43 burden seen by IHC is lower than in FTLD-TDP type A cases in the CA1 region^99^. FTLD-TDP type B cases also demonstrate diffuse NCIs and glial cell inclusions in the basal ganglia and substantia nigra^99,109^. As discussed above, FTLD-TDP type B cases also appear to involve loss of substance-P positive striatal efferents, with milder involvement of enkephalin-positive efferents. Furthermore, striatal involvement may be less severe in FTLD-TDP type B cases than in type A cases^108,111^. Of note, all FTLD-TDP type B cases demonstrating this apparent selective neuronal vulnerability presented clinically as FTD-MND^111^. 

 The thalamus appears to undergo minimal changes in most cases of FTLD-TDP type B. Only small numbers of thalamic NCIs tend to develop in type B cases^109^. Imaging studies demonstrate relatively minor focal atrophy in the lateral geniculate nucleus, ventral lateral, and mediodorsal nuclei of the thalamus^37^. *C9orf72 *repeat expansions however may predispose to more widespread thalamic atrophy, with greater involvement of the pulvinar^37,127^, but the exact cellular underpinnings of this change remain elusive. 

 Brainstem pathology is a core feature of ALS, and limited evidence suggests that involvement of the brainstem may be more widespread in FTLD-TDP type B than in type A or C^114,128,129^. In addition, involvement of cranial nerve motor nuclei is a well documented feature of FTLD-TDP type B^8^. FTLD-TDP type B often features glial cytoplasmic inclusions in the medulla, in addition to widespread neuronal alterations in the hypoglossal nucleus^23,99^. The latter are significantly more pronounced in FTLD-TDP type B than in other FTLD-TDP types. 

 Cerebellar TDP-43 pathology in FTLD-TDP is poorly documented, and the existing literature often does not stratify by histopathological subtype. Nonetheless, some clinically diagnosed cases of bvFTD or FTD-MND, with or without a *C9orf72 *repeat expansions, demonstrate mild but statistically significant atrophy in various regions of the cerebellum^115,130^. It is tempting to speculate that some of these cases may represent FTLD-TDP type B, but it is impossible to draw firm conclusions without autopsy confirmation. Histologically, no significant neuronal TDP-43 pathology or neurodegeneration has been seen in FTLD-TDP type B cases^99,131^, hence the molecular correlate substrate of cerebellar atrophy in FTLD-TDP type B remains unknown. 

Overall, current evidence suggests a selective vulnerability of cortical VENs, fork cells and motor neurons in FTLD-TDP type B. Preliminary data suggest that neocortical excitatory and calbindin-expressing neurons as well as substance-P positive striatal efferents are also affected. The selective neuronal vulnerability of other brain regions to TDP-43 pathology remains however largely unexplored. The relationship between TDP-43 pathology and loss of neuronal subtypes has only been established for VENs, fork cells and motor neurons.

#### 2.2.3 Glial involvement

 Astrocytic pathology has not been well characterized in FTLD-TDP type B. White matter pathology is less pronounced in FTLD-TDP type B than in type A, and most TDP-43 localizes to oligodendrocytes^117^. 

 In FTLD-TDP type B cases, activated microglia are distributed throughout all layers of the neocortex^43^. No significant differences have been found between sporadic FTLD-TDP and cases associated with a *C9orf72* mutation^43^. In contrast to FTLD-TDP types A and C, FTLD-TDP type B cases and controls show similar microglial burden as assessed by CD68, Iba1, or CR3/43, according to pairwise comparisons between cases and controls^42,43^. However, a trend towards increased CD68 reactivity was identified in the frontal white matter compared to controls^43^, suggesting that differences in microglial activation may have been overlooked in published yet underpowered studies. In conclusion, additional human studies are warranted to evaluate the involvement of macroglia and microglia in FTLD-TDP type B and their potential pathophysiological roles. 

### 2.3 FTLD-TDP type C

#### 2.3.1 General features

 FTLD-TDP type C accounts for 25 % of FTLD-TDP cases^8^. It is characterized by the presence of long dystrophic neurites, which tend to be concentrated in the superficial cortical laminae but are also present in deeper cortical layers^94,99^ (Figure 3). In FTLD-TDP type C there are few neuronal cytoplasmic inclusions and very few neuronal intranuclear inclusions^94,99^. Clinically, this form of FTLD usually manifests as svPPA, and sometimes also as nfvPPA or bvFTD^94^. 

#### 2.3.2 Neuronal pathology

 Type C cases typically demonstrate asymmetric cortical atrophy predominating in the temporal lobe^38^. In affected regions, superficial dystrophic neurites are visible on IHC, which are usually long and thick, in contrast to the shorter, comma-shaped dystrophic neurites of FTLD-TDP type A cases^94^. It is unclear which type of neuron is most vulnerable to the development of these inclusions. For instance, VENs have not been studied in a cohort enriched for FTLD-TDP type C cases. 

 In type FTLD-TDP type C cases, the dentate gyrus of the hippocampus exhibits compact NCIs that have been described as “Pick body-like” due to their uniformity^99^. The CA1 region does not generally contain NCIs, but does exhibit dystrophic neurites, which are usually not seen in FTLD-TDP type A or type B cases^99^. Notably, only a minority of cases demonstrate frank hippocampal sclerosis^109^. 

 Like the hippocampus, the striatum displays dystrophic neurites^99^ and compact NCIs with round contours in FTLD-TDP type C^99^. The nucleus accumbens appears to accumulate a greater pathological burden than the dorsal striatum, though the mechanism for this apparent regional selectivity is unknown^132^. Riku et al. found that two out of five FTLD-TDP type C cases displayed a selective degeneration of striatal efferents as described above for FTLD-TDP type A. Caution is however warranted given the low number of FTLD type C cases studied^111^. 

 In contrast to FTLD-TDP type A and type B cases, type C cases typically do not exhibit thalamic NCIs^109^. While one imaging study has found mild atrophy in the mediodorsal nucleus relative to controls, there was less thalamic atrophy than in any other pathological subtype of FTLD^37^. FTLD-TDP type C cases also exhibit only mild dystrophic neurites in the substantia nigra. As in type A, the hypoglossal nucleus is largely spared^99^. One study involving nine FTLD-TDP type C cases found no evidence of brainstem pathology in the midbrain, hypoglossal nucleus, or inferior olivary nucleus^109^, while another study involving five type C cases found TDP-43 deposits consistently in the superior colliculus, with occasional involvement of the inferior olivary nucleus and the red nucleus^114^. 

The cerebellum has not been systematically studied in FTLD-TDP type C. As discussed above for FTLD-TDP type A, some indirect evidence exists for cerebellar involvement FTLD-TDP in genera, but there have been no large studies examining differences between pathological subtypes, and the clinical significance of these findings remains unclear.

Unlike FTLD-TDP type A and B cases, FTLD-TDP type C cases appear to show concentrated TDP-43 pathology in the neocortex, hippocampus, and anterior striatum. It is surprising that the identity of vulnerable neurons that develop the characteristic long ‘corkscrew’ neurite is still unknown more than a decade after the formal recognition of this pathological FTLD subtype.

#### 2.3.3 Glial involvement

 It appears that glial inclusions, particularly subcortical white matter ones, are uncommon in FTLD-TDP type C^117^. It remains however unclear why TDP-43 does not tend to accumulate in cells of oligodendroglial morphology in FTLD-TDP type C cases, in contrast to the situation in types A and B. 

 Microglial activation has been studied to a significant degree in FTLD-TDP type C. Here, it appears to follow a similar pattern to that observed in type A cases, with more activation in the superficial cortical laminae and predominant white matter microgliosis in the frontal lobe^42^^,^^43^. Caution is however warranted, since the corresponding studies included only five^42^ and seven^43^ cases with FTLD-TDP type C pathology. 

These limited data suggest that macroglia are involved in FTLD-TDP type C in a less prominent manner than in other FTLD-TDP subtypes. Further studies evaluating astrocytic and microglial reactivity as well as oligodendroglial loss throughout the brain of FTLD-TDP type C cases could provide powerful insights into this question.

## 3. Frontotemporal lobar degeneration with fused in sarcoma (FUS) pathology

### 3.1 General features

 About five percent of FTLD cases exhibit neither tau nor TDP-43 pathology, and many of these cases are positive for FUS on IHC^8^. Three phenotypes of FUS-positive, TDP-43-negative, tau-negative FTLD have been described: atypical frontotemporal lobar degeneration with ubiquitin inclusions (aFTLD-U), basophilic inclusion body disease (BIBD), and neuronal intermediate filament inclusion disease (NIFID)^133^. 

 The most common subtype of FTLD-FUS, aFTLD-U, is characterized histologically by round, oval, or bean-shaped FUS-immunoreactive NCIs with a wide pattern of distribution^134^. These inclusions are also positive for Transportin 1, which mediates the nuclear import of FUS^135^. Additionally, aFTLD-U cases demonstrate long, thin, curved NIIs that have been described as “vermiform”^136^. 

 BIBD cases exhibit severe striatal and nigral atrophy, with varying degrees of cortical atrophy corresponding to behavioural symptoms^137,138^. The disease gets its name from basophilic cytoplasmic inclusions that are negative for neurofilament and tau but positive for FUS and occasionally positive for ubiquitin or p62^138^. Colocalization immunofluorescence has shown that FUS accumulates in inclusions identified by hematoxylin and eosin staining and inclusions identified by p62 IHC. In addition, FUS also accumulates in many more inclusions that could not be detected using other methods than IHC^138^. For this reason, BIBD is now generally classified as a FUS proteinopathy, though there are still many open questions about its pathogenesis and presentation. 

 NIFID cases also demonstrate involvement of various cortical and subcortical regions, and the observed NCIs and NIIs are heterogenous in their morphology and immunoreactivity^139^. The NCIs can be round, crescentic, annular, or tangle-like. In addition there are so-called “hyaline conglomerate inclusions” with a filamentous appearance and an eosinophilic core^140^. Both vermiform and round NIIs have been reported, and these appear to be more common in neurons that also exhibit round cytoplasmic inclusions^139,140^. These inclusions are immunoreactive for intermediate filament proteins and FUS, and colocalization of the latter has been confirmed using double-label immunofluorescence^139^. Nonetheless, some FTLD-FUS cases are immunoreactive for the intermediate filament alpha-internexin but not for FUS. Interestingly, one such case has been reported to exhibit both TDP-43 and alpha-internexin positivity^141^. 

 Clinically, aFTLD with ubiquitin inclusions (aFTLD-U) typically manifests early in life as an unusual form of bvFTD that can involve obsessions, pica, ritualistic behavior, and hypersexuality^133^. Due to their rarity, other forms of FTLD-FUS have not been characterized as systematically, but case reports suggest that they are also likely to present with early-onset bvFTD or FTD-MND^133,137,138,141,142^. While stereotyped and repetitive behaviours are reported in some cases, these symptoms are not as consistent as in aFTLD-U. Conversely, prominent MND and parkinsonism seem to be more common in both BIBD and NIFID^133,137,141^, and BIBD more commonly involves memory impairment and apraxia^133,137^. 

### 3.2 Neuronal pathology

 Cases of aFTLD-U demonstrate widespread NCIs throughout the frontal and temporal cortices^143^ and occasional NIIs in pyramidal neocortical neurons^14^. BIBD and NIFID cases also sometimes demonstrate neuronal inclusions in the frontal and temporal cortices^137^, but there are case reports of late-onset BIBD that preferentially affects the motor system, with minimal limbic or prefrontal involvement^143^. FTLD-FUS cases may also demonstrate selective involvement of VENs. While the sample size was small (*n=8*), one study found consistent and severe degeneration of GABRQ-positive neurons^104^. 

 Moderate to severe hippocampal involvement is a common feature in aFTLD-U, with FUS-positive NCIs and vermiform NIIs in the granule cells of the dentate gyrus and, to a lesser extent, in the subiculum and CA1 regions^14,133^. In contrast, BIBD demonstrates less consistent involvement of the dentate granule cells and more consistent involvement of the pyramidal neurons^134,136,138^. Hippocampal inclusions can also be found in NIFID^140,144^, but in at least a subset of cases, inclusions in the hippocampus are not nearly as numerous as in the frontal lobe^144^. 

 Severe striatal atrophy has been reported in all three major subtypes of FTLD-FUS^136^. In aFTLD-U, there are often crescentic NCIs and small numbers of NIIs^136^, and the striatum can exhibit varying degrees of involvement^14,143,145^. In BIBD, severe striatal atrophy is a consistent finding, and in some cases, striatal pathology is considerably more severe than cortical or hippocampal pathology^138^. Both caudate and putamen demonstrate severe neuronal loss, gliosis, and basophilic inclusions^138^, and early involvement of the striatum and the pyramidal motor system are more common in BIBD than in aFTLD-U^143^. As in BIBD, NIFID cases are thought to show severe, consistent striatal atrophy^136,137^, though a recent case series has presented numerous cases without evidence of atrophy^141^. The globus pallidus^136^ is also involved in FTLD-FUS, particularly in BIBD and NIFID but also in aFTLD-U^14,136^. However, some cases appear to exhibit only mild pallidal atrophy despite significant involvement of the striatum^133,137^, and there are some cases of aFTLD-U that involve extreme caudate atrophy with relative preservation of both putamen and globus pallidus^133^. 

 In aFTLD-U cases, the thalamus is not a consistent site of pathology, but many cases show small numbers of NCIs and NIIs^136,140^. In contrast, BIBD appears to consistently demonstrate moderate to severe pathological burden in the thalamus, including non-compact collections of coarse granules and even occasional NIIs^140^. Finally, NIFID also shows relatively consistent involvement of the thalamus, though the level of pathology can vary considerably^136,140,144^. 

 The brainstem also seems to be involved in aFTLD-U, with greater involvement of rostral regions^143^. However, many cases demonstrate only mild FUS-immunoreactive pathology in the pons and midbrain, including the substantia nigra but sparing the red nucleus^140^. In BIBD, there are basophilic, FUS-immunoreactive inclusions in the brainstem^136^, particularly in the pontine nuclei and the inferior olivary nucleus^136^. Indeed, along with the basal ganglia, the brainstem appears to be among the regions with the highest density of FUS-positive inclusions in BIBD^137^. Caudal regions of the brainstem display more pronounced pathology than rostral regions in BIBD^143^. Furthermore, lower motor neurons in the spinal cord are affected^136,137^. NIFID also shows more consistent and widespread brainstem pathology than aFTLD-U, including involvement of the locus coeruleus, the red nucleus, and the basis pontis^140^, though gross atrophy is not always apparent^144^. Much of the brainstem pathology presents as coarse granules rather than as compact inclusions^139^. 

 Cerebellar involvement appears to be an uncommon finding in aFTLD-U, though some cases exhibit a small number of NCIs in the dentate nucleus^136^. Cerebellar involvement seems to be somewhat more common in BIBD, with NCIs and GCIs often present in the dentate nucleus^137,138,143^. Nonetheless, the cerebellar cortex is not generally affected^143^. NIFID cases also sometimes demonstrate neuronal loss and FUS-immunoreactive pathology in the cerebellum^136,139^. However, this does not appear to be a consistent finding^144^, and there are fewer FUS-immunoreactive inclusions in the cerebellum than in virtually any other grey matter region that has been subjected to study^134^. 

In summary, the distribution of FUS-immunoreactive pathology has been described in all three types of FTLD-FUS. However, only VENs and GABRQ+ neuronal subtypes have been proven to be affected by the pathological inclusions. Further subtype-specific studies of neuronal loss and correlative studies with FUS inclusions in each FTLD-FUS subtype are needed to understand the pathogenic mechanisms of disease in these entities.

### 3.3 Glial involvement

 All three major forms of FTLD-FUS are associated with glial cytoplasmic inclusions in the white matter^14,134,139,140^. These inclusions are typically oval or flame-shaped^134^. Similar inclusions have been identified in ALS with a FUS mutation^145^. Double labelling has demonstrated that the inclusions localize to oligodendrocytes^145^. Based on these findings and the morphological characteristics of the involved cells in FTLD-FUS, it seems reasonable to conclude that the GCIs represent pathological involvement of oligodendrocytes. The distribution and protein expression of affected cells have however not been systematically studied. 

 In one study comparing microglial activation across subtypes of FTLD, FTLD-FUS cases did not appear to demonstrate greater grey matter microglial activation than controls. Yet, only four cases were included, and all cases were aFTLD-U^42^. In the white matter, there appears to be a possible trend toward increased microglial activation compared to controls in frontal and temporal white matter, but a statistically significant difference has not been demonstrated^42^. 

## Conclusion

There is clear evidence for selective region- and cell-type specific vulnerability in all forms of FTLD, which helps explain the distinct clinical and pathological features associated with each subtype. Where there is evidence of selective neuronal vulnerability, such as VENs in PiD and FTLD-TDP or striatal substance-P-positive efferents in FTLD-TDP, further characterization of the changes to those populations and comparisons to unaffected neuronal types would be of value. Where there is little evidence on selectively vulnerable populations, such as in FTLD-FUS, systematic attempts to identify and characterize vulnerable cell types could help focus further research. As the scientific community has learned from attempts to understand other neurodegenerative conditions, abnormal protein inclusions are excellent markers for disease classification and diagnosis but are unlikely to be the sole pathophysiological factor at play. This highlights the importance of systematic studies to understand selective vulnerability of both neurons and glia and the precise distribution of these cell types in FTLD. When a selectively vulnerable population is identified, exploration of associated glia, synaptic inputs, and projections can provide mechanistic information beyond that afforded by characterization of isolated affected neurons.

This review suggests a number of areas for further research, including the evaluation of neuronal subtype-specific vulnerability; the significance and distribution of oligodendroglial and astrocytic pathology; the relationship between activated microglia and vulnerable neuronal populations; and the relative importance of cell-autonomous versus non-cell-autonomous mechanisms of neurodegeneration. This latter aspect has not been extensively evaluated in humans. Closing the knowledge gap on some of these areas may identify pathological processes amenable to therapeutic intervention, which will likely be disease specific as the existing literature suggests.

## Conflict of interest statement

The author declares no conflict of interest.
